# Predicting the future of excitation energy transfer in light-harvesting complex with artificial intelligence-based quantum dynamics

**DOI:** 10.1038/s41467-022-29621-w

**Published:** 2022-04-11

**Authors:** Arif Ullah, Pavlo O. Dral

**Affiliations:** State Key Laboratory of Physical Chemistry of Solid Surfaces, Fujian Provincial Key Laboratory of Theoretical and Computational Chemistry, Department of Chemistry, and College of Chemistry and Chemical Engineering, Xiamen University, Xiamen, 361005 Fujian China

**Keywords:** Chemical physics, Quantum mechanics, Computational chemistry, Method development

## Abstract

Exploring excitation energy transfer (EET) in light-harvesting complexes (LHCs) is essential for understanding the natural processes and design of highly-efficient photovoltaic devices. LHCs are open systems, where quantum effects may play a crucial role for almost perfect utilization of solar energy. Simulation of energy transfer with inclusion of quantum effects can be done within the framework of dissipative quantum dynamics (QD), which are computationally expensive. Thus, artificial intelligence (AI) offers itself as a tool for reducing the computational cost. Here we suggest AI-QD approach using AI to directly predict QD as a function of time and other parameters such as temperature, reorganization energy, etc., completely circumventing the need of recursive step-wise dynamics propagation in contrast to the traditional QD and alternative, recursive AI-based QD approaches. Our trajectory-learning AI-QD approach is able to predict the correct asymptotic behavior of QD at infinite time. We demonstrate AI-QD on seven-sites Fenna–Matthews–Olson (FMO) complex.

## Abstract (web summary)

Simulations of energy transfer in light-harvesting complexes are computationally very demanding. Here the authors apply an artificial intelligence quantum dissipative algorithm to study the excited state energy transfer dynamics in a light-harvesting complex.

## Introduction

From the birth of life, solar energy has been the driving force of life. Via the mechanism of photosynthesis, living organisms capture sunlight with the highly sophisticated pigments in their antenna systems and transfer sunlight energy to the reaction center (RC) in the form of electron-hole pairs (excitons), where it is stored as biochemical energy^1^. The transfer of solar energy from antenna to RC, which is also known as excitation energy transfer (EET), in the form of excitons is considered to be highly efficient with close to unit efficiency^2^. Understanding this high efficiency of the natural harvesting systems is very important because this understanding can be potentially applied in designing very efficient organic solar cells and storage devices^3^. Experiments showed that the long-lasting coherence in the efficient natural light-harvesting complexes (LHCs) is preserved by the surrounding protein environments (scaffold), and this coherence may be responsible for this high efficiency^4,5^. The most well-investigated LHC is Fenna–Matthews–Olsen (FMO) complex, which is found in green sulfur bacteria^6^. The small size and simplicity of the FMO complex also make it a testbed of simulation approaches. The FMO complex is a trimer of identical subunits, where each subunit consists of bacteriochlorophyll (BChl) molecules (system) attached to their protein environments^7^.

Enormous amount of research work has been done on light-harvesting processes^8–13^. Taking FMO as an example, it is easy to see that the system (BChl molecules) is not isolated from the environment (the protein) and thus, the correct simulation of FMO should treat it as an open system rather than isolated one. In addition, many experiments suggest^14,15^, that quantum effects, particularly coherence, might play an important role in the light-harvesting processes and may even be responsible for achieving the high-end efficiency. Temporal and spatial simulation of EET with the inclusion of quantum effects can be done within many frameworks such as classical mapping-based approaches^16–18^, perturbative methods^19–21^, and dissipative quantum dynamics (QD)^22–27^ adopted here.

QD simulations can be performed using the hierarchical equations of motion (HEOM)^28^ and its many improvements and extensions^8,23,29–31^, the quasiadiabatic propagator path integral (QuAPI)^32^ and its variant iterative QuAPI (iQuAPI)^27^, the trajectory-based stochastic equation of motion (SEOM) approach^25,33–39^, the multi-layer multi-configuration time-dependent Hartree (ML-MCTDH)^26^ and the local thermalising Lindblad master equation (LTLME)^22^. The development of various quantum dissipative dynamics methods stirs from the fact that each of these methods has some limitations and hence there is no single universal method that works in all cases. For instance, HEOM is numerically exact but comes with a very high computational cost at low temperatures, the SEOM has no explicit dependence on the temperature but has very bad convergence at long-time propagation, in the QuAPI approach all correlation effects are included over a finite time and correlation effects beyond this time are neglected. Most importantly, all these traditional QD approaches require step-wise propagation of trajectories and the next step depends on the previous steps, thus, QD simulation is an iterative, recursive process. Both calculations at each time step and recursive nature of QD makes it rather computationally expensive.

Alleviating the computational cost of QD became a target of a series of studies applying artificial intelligence (AI)^40–46^, inspired by advances in application of AI employing machine learning (ML) algorithms in computational chemistry and chemical physics^47,48^. AI was also applied to investigate EET in a dimer system^44^ and the FMO complex^40^. Saving of computational cost by AI in above studies is impressive, however, one of the studies^40^ only focused on predicting energy transfer times and transfer efficiencies rather than temporal and spatial evolution, while other related studies^44–46^ adopted basically the same recursive nature of QD trajectory propagation.

The recursive nature of the previous AI-based QD makes it prone to error accumulation. In recursive simulations, previously predicted values are used as an input to predict the next value. Thus, the prediction error at each time-step will accumulate, which results in deterioration of accuracy. In addition, the recursive nature of predictions does not allow us to make a prediction for any arbitrary time without predicting values before that. Finally, a short-time trajectory is needed as the seed to be generated with traditional approaches such as HEOM and then provided as an input to AI model to make prediction for the next time step and ultimately propagate the long-time dynamics. Thus, even when having AI model, we still need to spend valuable computational time to generate the short-time trajectory with the traditional approaches.

Here, we suggest an AI-QD approach to directly predict QD with AI as a function of time and other parameters such as temperature, reorganization energy, etc., completely circumventing the need of recursive step-wise dynamics propagation in contrast to the traditional QD and alternative, recursive AI-based QD approaches. Our AI-QD approach is able to predict QD at infinite time with correct asymptotic behavior and can be viewed as trajectory learning, which does not need any short-time trajectory as an input, eradicates the need of traditional approaches to generate the seed, and alleviates the problem of error accumulation. We demonstrate the applicability of AI-QD on seven-sites Fenna–Matthews–Olson (FMO) complex and show how AI-QD can be used for massive, infinite-time QD simulations and provide insights into the desired range of parameters and more efficient paths followed by the transfer of excitation energy.

## Results

### Reference quantum dynamics of the FMO complex

We employ the Frenkel exciton Hamiltonian^49^ to study EET dynamics in the FMO complex:

1 $${{{{{{{\bf{H}}}}}}}}={{{{{{{{\bf{H}}}}}}}}}_{{{{{{{{\rm{s}}}}}}}}}+{{{{{{{{\bf{H}}}}}}}}}_{{{{{{{{\rm{env}}}}}}}}}+{{{{{{{{\bf{H}}}}}}}}}_{{{{{{{{\mbox{s-env}}}}}}}}}+{{{{{{{{\bf{H}}}}}}}}}_{{{{{{{{\rm{reorg}}}}}}}}},$$

with all Hamiltonian terms given below

2 $${{{{{{{{\bf{H}}}}}}}}}_{{{{{{{{\rm{s}}}}}}}}}=\mathop{\sum }\limits_{i}^{n}\left|i\right\rangle {\epsilon }_{i}\left\langle i\right|+\mathop{\sum }\limits_{i,j = 1,i\ne j}^{n}\left|i\right\rangle {J}_{ij}\left\langle j\right|,$$

3 $${{{{{{{{\bf{H}}}}}}}}}_{{{{{{{{\rm{env}}}}}}}}}=\mathop{\sum }\limits_{i=1}^{n}\mathop{\sum}\limits_{k=1}\left(\frac{1}{2}{{{{{{{{\bf{P}}}}}}}}}_{k,i}^{2}+\frac{1}{2}{\omega }_{k,i}^{2}{{{{{{{{\bf{Q}}}}}}}}}_{k,i}^{2}\right),$$

4 $${{{{{{{{\bf{H}}}}}}}}}_{{{{{{{{\mbox{s-env}}}}}}}}}=-\mathop{\sum }\limits_{i=1}^{n}\mathop{\sum}\limits_{k=1}\left|i\right\rangle {c}_{k,i}{{{{{{{{\bf{Q}}}}}}}}}_{k,i}\left\langle i\right|,$$

5 $${{{{{{{{\bf{H}}}}}}}}}_{{{{{{{{\rm{reorg}}}}}}}}}=\mathop{\sum }\limits_{i=1}^{n}\left|i\right\rangle {\lambda }_{i}\left\langle i\right|,$$

where **H**_s_, **H**_env_, **H**_s-env_, and **H**_reorg_ denote system (BChl molecules) Hamiltonian, Hamiltonian of protein-environment, system-environment interaction Hamiltonian and the reorganization term, respectively. In Eq. (1), *n* is the number of sites (BChl molecules), *ϵ*_*i*_ is the energy of the *i*th site and *J*_*ij*_ is the inter-site coupling between sites *i* and *j*. **P**_*k*,*i*_,  **Q**_*k*,*i*_, and *ω*_*k,i*_ are, respectively, momentum, coordinate, and frequency of environment mode *k* associated with site *i*. In **H**_s-env_, each site is connected to its own environment. The *c*_*k*,*i*_ is the strength of coupling between site *i* and mode *k* of its environment. The reorganization term **H**_reorg_ can be seen as a counter term that emerges from the interaction of the sites with the environment^8,49,50^. It is added to stop further renormalization of the site energy *ϵ*_*i*_ by the environment. In the reorganization term **H**_reorg_, *λ*_*i*_ is the reorganization energy corresponding to site *i*^51^,

6 $${\lambda }_{i}=\frac{1}{\pi }\int\nolimits_{0}^{\infty }\frac{{J}_{i}(\omega )}{\omega }d\omega ,$$

where *J*_*i*_(*ω*) is spectral density of the environment corresponding to site *i*. As shown by Nalbach and Thorwart^52^, the effects of the discrete molecular modes on the population dynamics are largely irrelevant. As a result, it is acceptable to use continuous environment spectral density such as Drude–Lorentz spectral density

7 $${J}_{{{\mbox{env}}}}(\omega )=2\lambda \frac{\omega \gamma }{{\omega }^{2}+{\gamma }^{2}},$$

where *γ* and *λ* denote the characteristic frequency (bath relaxation rate) and the reorganization energy, respectively.

In general terms, the EET dynamics in the FMO complex can be described by Liouville–von Neumann equation

8 $$\frac{d}{dt}{{{{{{{\boldsymbol{\rho }}}}}}}}(t)=\frac{i}{\hslash }\left[{{{{{{{\bf{H}}}}}}}},{{{{{{{\boldsymbol{\rho }}}}}}}}(t)\right],$$

where ***ρ*** is the density matrix. Because of the many-body effects, direct propagation of Eq. (8) is not straightforward. Different approaches are developed to simplify and propagate Eq. (8) and interested readers are advised to look into the corresponding references^25,30,32,53^.

We use the local thermalising Lindblad master equation (LTLME)^22^ to propagate the reference QD trajectories for the reduced density matrix of the system (see Supplementary Methods), where we adopt Adolphs and Renger’s Hamiltonian for seven sites per subunit^54^ (see “Methods”). The LTLME is a coherent and complete positive trace-preserving approach, but may not be as accurate as HEOM or SEOM approaches because of approximations used in LTLME derivation^22,55^, but here it is not the concern of our proof-of-concept paper.

### Parameters-based non-recursive training framework

**Fig. 1:**
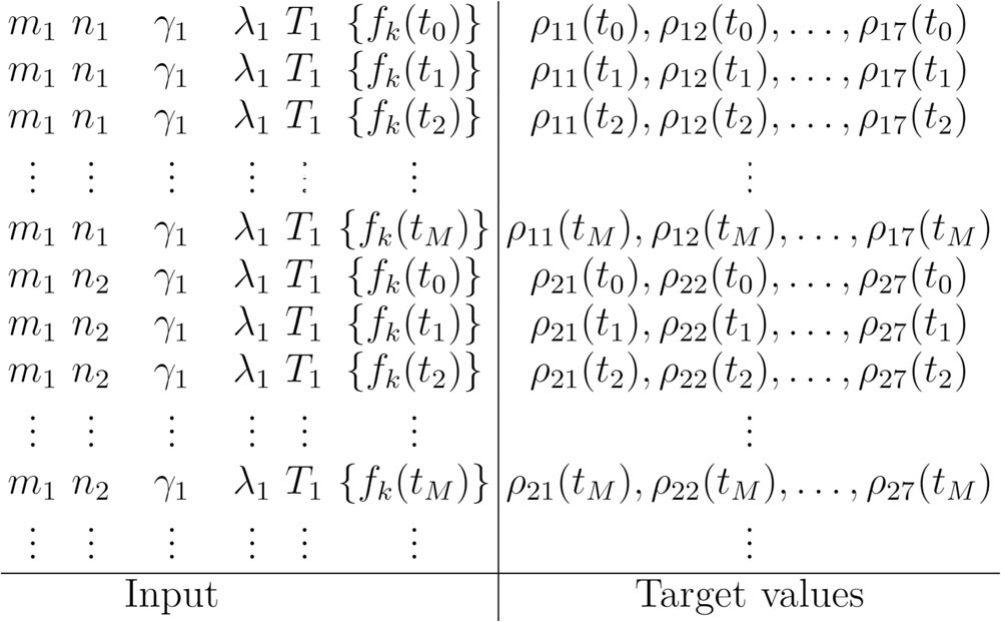
Preparation of training data using parameters in AI-QD training framework. Here $\left\{{f}_{k}(t)\right\}$ is a set of time-functions based on the logistic function ${f}_{k}(t)=1/(1+15\cdot \exp (-(t+{c}_{k})))$ where *c*_*k*_ = 5*k*−1.0 and *k* ∈ {0, 1, 2, …, 99} (see “Methods”). Other parameters are *t* = {*t*_0_, *t*_1_, *t*_2_, …, *t*_*M*_}, *λ* = {*λ*_1_, *λ*_2_, *λ*_3_, …, *λ*_*i*_}, *γ* = {*γ*_1_, *γ*_2_, *γ*_3_, …, *γ*_*j*_}, and *T* = {*T*_1_, *T*_2_, *T*_3_, …, *T*_*l*_}. In addition, labels *n* = {*n*_1_, *n*_2_, *n*_3_, …, *n*_7_} are used for corresponding rows in the density matrix and labels for sites with possible initial excitation are *m* = {*m*_1_, *m*_2_}. As the off-diagonal elements *ρ*_*nq*, *n*≠*q*_ are complex, we separate the real and imaginary parts.

In our parameters-based non-recursive AI-QD, we train ML model as a function of a parameter space ${{{{{{{\mathcal{D}}}}}}}}$ (used as the input to ML model) which depends on the system of interest and on the data from a limited number of QD trajectories. For the FMO complex, our parameter space ${{{{{{{\mathcal{D}}}}}}}}$ consists of information of sites: *λ*, *γ*, and *T*. In addition, time also becomes a part of the input of our AI-QD model. In order to treat infinite time, instead of time, we introduce time-function $f(t)\in {{{{{{{\mathcal{D}}}}}}}}$, which normalizes time and for *t* → *∞* becomes *f*(*t*) = 1. Such normalization, however, can effectively only discern data within rather short time-region, thus, instead of a single time-function, we introduce the set of redundant time-functions $\left\{{f}_{k}(t)\right\}$ for different regions in very long-time propagation (see “Methods”). The remaining input of our model is information about the initial excitation $m=\{{m}_{1},{m}_{2}\}=\{0,1\}\in {{{{{{{\mathcal{D}}}}}}}}$ (with 0 corresponding to initial excitation on site-1 and 1 corresponding to site-6) and labels $n=\{{n}_{1},{n}_{2},{n}_{3},\ldots, {n}_{7}\}=\{1,2,3,\ldots ,7\}\in {{{{{{{\mathcal{D}}}}}}}}$ corresponding to the seven rows in the reduced density matrix. We train convolutional neural network (CNN) taking all above input elements $\{m,n,\,\gamma ,\,\lambda ,\,T,\,\{f_k(t)\}\}\in {{{{{{{\mathcal{D}}}}}}}}$ on rows of the reduced density matrix which include exciton population *ρ*_*nn*_(*t*) and coherence (off-diagonal) terms *ρ*_*nq, n≠q*_ (target values to learn or output of the trained model) (see Fig. 1 and “Methods” for details, such as CNN architecture and normalization of input elements).

Our training trajectories generated with the reference LTLME-QD approach are chosen by farthest-point sampling from the three-dimensional space of the following parameters: reorganization energy *λ* = {*λ*_1_, *λ*_2_, *λ*_3_, …, *λ*_*i*_}, the characteristic frequency *γ* = {*γ*_1_, *γ*_2_, *γ*_3_, …, *γ*_*j*_} and temperature *T* = {*T*_1_, *T*_2_, *T*_3_, …, *T*_*l*_} (see “Methods”).

We should also decide up to what time-length *t*_*M*_ we should run reference LTLME-QD trajectories. Based on the prior knowledge that populations plateau in asymptotic limit, for each trajectory we choose a different time-length *t*_*M*_ using a vanishing gradient scheme, where *t*_*M*_ is chosen such that the gradient of population *G* is close to zero (see “Methods”). Using the vanishing gradient scheme to find different *t*_*M*_ for each trajectory allows us to sample more data from the training trajectories, which are hard-to-learn, while avoiding redundant sampling from trajectories, which are easy-to-learn. This also removes arbitrariness in choosing fixed *t*_*M*_ parameter as was done in previous studies using the recursive AI-QD scheme^44,46^.

### Application to EET dynamics in FMO complex

**Fig. 2:**
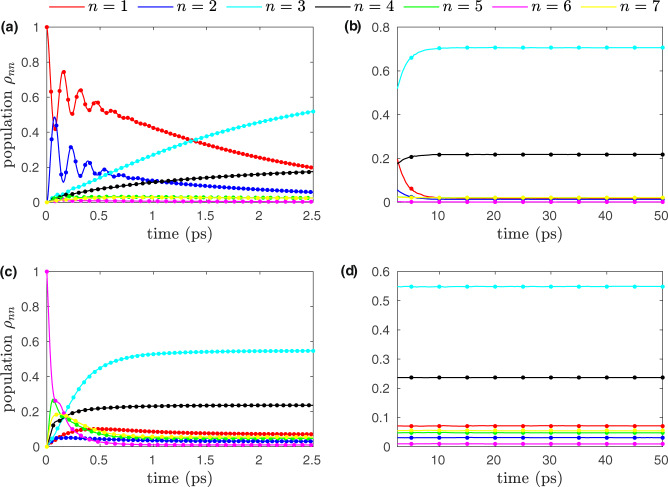
Population of the seven sites in the FMO complex as a function of time. In **a**, **b**, the initial excitation is considered on site-1 and other parameters are *γ* = 175, *λ* = 70, *T* = 70. In **c**, **d**, the initial excitation is on site-6 and other parameters are *γ* = 75, *λ* = 100, *T* = 130. **a** and **c** show a part of the population up to 2.5 ps, while the population changes beyond 2.5 ps are shown in (**b**) and (**d**), from which it is clearly seen that the population plateaus after a few picoseconds. The off-diagonal terms or coherences are shown in Fig. 3. The results of AI-QD are compared to the results of LTLME-QD (dots). *n* is the site label. *γ* and *λ* are in the units of cm^−1^, while *T* is in the units of K.

**Fig. 3:**
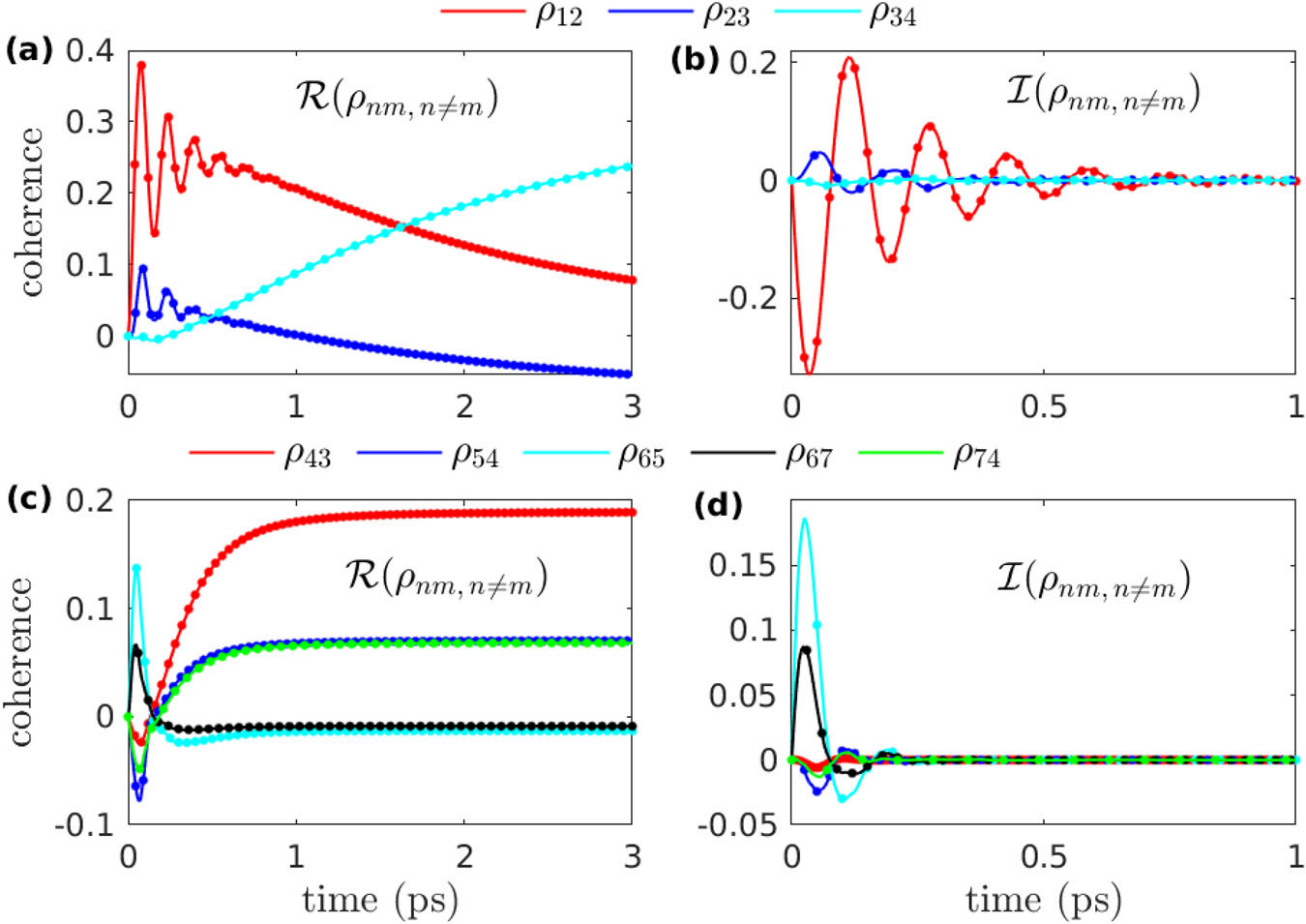
Electronic coherence as a function of time. **a** and **b**, respectively, show the real and imaginary parts of the prominent off-diagonal terms for Fig. 2a, b, where *γ* = 175, *λ* = 70, *T* = 70 with the initial excitation on site-1. **c** and **d**, respectively, show the real and imaginary part of the prominent off-diagonal terms for Fig. 2c, d, where *γ* = 75, *λ* = 100, *T* = 130 with the initial excitation on site-6. The results of AI-QD are compared to the results of LTLME-QD (dots). *γ* and *λ* are in units of cm^−1^, while *T* is in the units of K.

As an application of our approach, we predict EET dynamics in the FMO complex with seven sites per subunit for parameters of the test set trajectories none of which used in training. Site-1 (BChl molecule 1) and site-6 (BChl molecule 6) are most likely to get initially excited as they are close to the photosynthetic antenna complex called chlorosome^6^, we thus present results for both cases. For predictions, we just provide the parameters of the test trajectories (characteristic frequency, reorganization energy, temperature) as an input and predict the evolution of EET. Figure 2 shows the evolution of excitation energy in all seven sites for both cases. In Fig. 2, we show EET for both short and long time periods, demonstrating that AI-QD is able to capture the coherent EET (aka quantum beating or modulation of amplitudes) of short-time dynamics and also can predict the asymptotic limit. Figure 3 shows the prominent off-diagonal terms (aka coherence) of the reduced density matrix for Fig. 2. Table 1 shows mean absolute error (MAE) and root mean square error (RMSE) averaged over 600 trajectories. As AI-QD is non-recursive (non-iterative), without any trajectory propagation, we can directly predict the asymptotic behavior. Our AI-QD performs well in all cases (from weak coherence to strong coherence, from Markovian to non-Markovian, from adiabatic to nonadiabatic situations) as can be observed for selected trajectories shown in Supplementary Fig. 1 with corresponding errors reported in Supplementary Table 1. From Supplementary Table 1, we observe that our AI-QD approach is comparatively more accurate in strongly coherent cases (large value of *γ* and small values of *λ* and *T*) which can be seen as a consequence of the vanishing gradient scheme which may favor these challenging cases due to a larger number of training points sampled from such trajectories. AI-QD approach can even extrapolate to a good degree as its error for the test trajectories propagated with parameters outside the training parameter space is of a similar order of magnitude to the test trajectories propagated with parameters inside the training parameter space (interpolation) as shown in Supplementary Fig. 2 and Supplementary Table 2.

**Table 1:** Mean absolute error (MAE) and root mean square error (RMSE) averaged over 600 test trajectories propagated up to 1 ns.

	Diagonal terms	Off-diagonal terms	
Error	*ρ*_*nn*_	${{{{{{{\mathcal{R}}}}}}}}\{{\rho }_{mn,n\ne m}\}$	${{{{{{{\mathcal{I}}}}}}}}\{{\rho }_{mn,n\ne m}\}$
MAE	1.3 × 10^−3^	5.1 × 10^−4^	2.4 × 10^−4^
RMSE	2.1 × 10^−3^	8.1 × 10^−4^	3.6 × 10^−4^

${{{{{{{\mathcal{R}}}}}}}}\{{\rho }_{mn,n\ne m}\}$ and ${{{{{{{\mathcal{I}}}}}}}}\{{\rho }_{mn,n\ne m}\}$ represent the real and imaginary part of the off-diagonal terms, respectively.

**Fig. 4:**
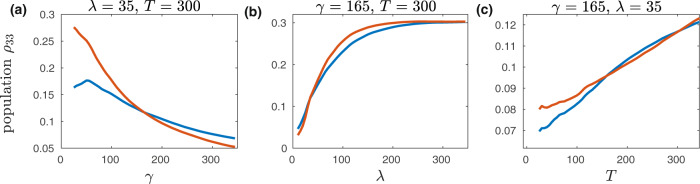
The evolution of site-3 population at *t* = 0.5 ps. Plots are shown as a function of (**a**) characteristic frequency of the environment *γ* (**b**) reorganization energy *λ*, and **c** temperature *T*. The blue line corresponds to the case with initial excition on site-1 while the red line is for the case with initial excition on site-6. *γ* and *λ* are in the units of cm^−1^ while *T* is in the units of K.

It was shown^8,56,57^ that the transfer of excitation energy in the seven-sites FMO complex follows mainly two paths, i.e., site-1 → site-2 → site-3 ↔ site-4 and site-6 → site-5, site-7, site-4 → site-3, here the ↔ shows that the excitation energy equilibrates between site-3 and site-4 after site-3 is populated (see Fig. 3). Among the seven sites, the sites 1 and 6 are close to the baseplate protein, while the sites 3 and 4 are near to the target RC complex^54,58^. It has been proposed that the quantum coherence allows the FMO complex to quickly sample several routes (paths) in search of site-3^5^. In Fig. 4, we show the population of site-3 at *t* = 0.5 ps (500 fs) as a function of *γ*, *λ*, and *T*. From Fig. 4a, we observe that at room temperature *T* = 300, the ETT to site-3 or, in other words, to RC complex gets slow as the characteristic frequency *γ* increases. In contrast, the ETT to site-3 increases with the increase in reorganization energy *λ* as shown in Fig. 4b. Similar trend can be observed with the increase in temperature *T* as can be seen in Fig. 4c.

In order to find the optimum parameters for the fastest transfer of excitation energy, we have calculated population of site-3 at 0.5 ps for a massive set of ca. 0.57 million possible combinations (site-1 + site-6) of the *γ*, *λ*, *T* with the search space *γ* = 25, 30, 35, …, 245, *λ* = 10, 15, 20, …, 345 and *T* = 25, 30, 35, …, 345. We report the fastest EET of 0.761 to site-3 for path-2 with *γ* = 30, *λ* = 310, *T* = 25, while for path-1 for the same parameters EET is 0.626. From Figs. 2, 4 and from the optimum parameters, we notice that following path-1, i.e., site-1 → site-2 → site-3 ↔ site-4, the EET shows more coherence and is slow compared to excitation transfer following path-2, i.e., site-6 → site-5, site-7, site-4 → site-3. From Eq. (9) (“Methods”), energy of the site-1 (12,410 cm^−1^) is lower than the baseplate, which has been reported to be 12,500 cm^−1 ^^59,60^. This allows a quick transfer of the excitation energy to site-1 from the baseplate. However, the energy of site-2 (12,530 cm^−1^) is higher than site-1 and also than site-3 (12,210 cm^−1^), which on the one hand stops backward transfer from site-3, but on the other hand creates a local minimum on site-1. Despite the local minimum on site-1, the excitation energy is not trapped because of the quantum coherent wave-like motion between site-1 and site-2. Following path-2, the energy of site-6 (12,630 cm^−1^) is higher than the energy of baseplate. To stop backward transfer of excitation energy from site-6 to baseplate, site-6 should quickly transfer excitation energy to other sites such as site-5, site-7, and site-4. This quick transfer from site-6 to site-5, site-7, and site-4 is only possible by the strong coupling of site-6 to site-5 and site-7, which in return are strongly coupled to site-4.

## Discussion

In this work, we have presented a non-recursive (non-iterative) AI-QD approach for blazingly fast prediction of quantum dynamics, as predictions can be made for any time step up to asymptotic limit completely circumventing the need of recursive trajectory propagation. This can be used, as we demonstrated here, for massive quantum dynamics simulations, for example, in search for the best conditions required for efficient energy transfer in designed photovoltaic devices. Just to put things into perspective, our AI-QD approach can predict the entire 2.5 ps trajectory within ca. 2 min on a single core of Intel(R) Core(TM) i7-10700 CPUs @ 2.90 GHz, independent of the reference method used for generating training trajectories, while the same propagation with the traditional recursive approaches such as HEOM would take hours, and the cost would exponentially increase for low temperatures. The high cost of accurate approaches such as HEOM was also a reason why we used a much faster LTLME for this proof-of-concept study to extensively test our approach (propagation of an entire trajectory takes only 3 min with LTLME on a single CPU of the above computer architecture). It is worth emphasizing that AI-QD is embarrassingly parallel and the calculations can be further significantly sped up by using multiple CPUs or GPUs, because predictions with AI-QD for different time steps are independent of each other and different segments of trajectories can be distributed for independent calculations on many threads.

We demonstrated the feasibility of AI-QD approach on an example of the FMO complex, but this approach is general enough to be used for any other complex after retraining. It remains to be seen how well the AI-QD approach can be extended to describe several LHCs at the same time—a topic of our ongoing research. One could use the LHC Hamiltonian elements as a representation of LHC complexes and an early encouraging study^42^ has shown that by using Hamiltonian elements as input of an ML model, one can successfully describe scalar properties (energy transfer times and transfer efficiencies) for different Hamiltonians. However, open question remains how successful would be such an approach to learn dynamics and in addition, how to circumvent different dimensionalities of Hamiltonians of different complexes.

## Methods

### Training data

In the seven-sites FMO complex (apo-FMO), where seven BChl molecules (seven sites) exist per subunit, the inter-subunit interaction is very small and each subunit can be considered relatively isolated^61^. Here we adopt Adolphs and Renger’s Hamiltonian for seven sites per subunit^54^

9 $${{{{{{\bf{H}}}}}}}_{{{{{{\rm{s}}}}}}}=\left[\begin{array}{ccccccc}12410&-87.7&5.5&-5.9&6.7&-13.7&-9.9\\ -87.7&12530&30.8&8.2&0.7&11.8&4.3\\ 5.5&30.8&12210&-53.5&-2.2&-9.6&6.0\\ -5.9&8.2&-53.5&12320&-70.7&-17.0&-63.6\\ 6.7&0.7&-2.2&-70.7&12480&81.1&-1.3\\ -13.7&11.8&-9.6&-17.0&81.1&12630&39.7\\ -9.9&4.3&6.0&-63.3&-1.3&39.7&12440\end{array}\right],$$

where energies are given in cm^−1^. Each site is coupled to its own environment characterized by the Drude–Lorentz spectral density given by Eq. (7). Not long ago, an eighth BChl molecule (site-8) has been discovered^11^, however, as has been mentioned by Jia et al.^62^, the role of the eighth BChl molecule (site-8) in the transfer of excitation energy in the FMO complex is negligible.

Trajectories for the reduced density matrix have been generated with the local thermalising Lindblad master equation (LTLME)^22^ (see Supplementary Methods) implemented in quantum_HEOM package^63^ with QuTip^64^ in the back-end with all the possible combinations of the following parameters: *λ* = {10, 40, 70, 100, 130, 160, 190, 220, 250, 280, 310} cm^−1^, *γ* = {25, 50, 75, 100, 125, 150, 175, 200, 225, 250, 275, 300} cm^−1^ and *T* = {30, 50, 70, 90, 110, 130, 150, 170, 190, 210, 230, 250, 270, 290, 310} K. We consider that all these combinations of parameters make a part of a parameter space ${{{{{{{\mathcal{D}}}}}}}}$. The time-step used for propagation is 5 fs and the trajectory is propagated up to *t*_*M*_ = 1 ns (10^6^ fs). With the possibility of initial excitation on site-1 and site-6, we generate 1980 trajectories for each excitation case.

### Data preparation

With all the possible combinations of the parameters *λ*, *γ*, *T* (belonging to ${{{{{{{\mathcal{D}}}}}}}}$), we have 3960 total number of trajectories N_traj_ (1980 (site-1) + 1980 (site-6), all these trajectories correspond to their respective combination of parameters in parameter space ${{{{{{{\mathcal{D}}}}}}}}$). Using farthest-point sampling^65^ in the three-dimensional space of *λ*, *γ*, and *T*, we choose 1000 trajectories as our training space TS (500 (site-1) + 500 (site-6), ca. 25% of space ${{{{{{{\mathcal{D}}}}}}}}$)), 200 trajectories as the validation set VS (ca. 5% of space ${{{{{{{\mathcal{D}}}}}}}}$)) and the rest of trajectories, we keep as the test set STP (set of test points, ca. 70% of space ${{{{{{{\mathcal{D}}}}}}}}$). For each trajectory, we choose a different time-length *t*_*M*_ using a vanishing gradient scheme. In this scheme, we take the gradient *G* of the population of each site (*ρ*_*nn*_, *n* = 1, 2, 3, …, 7) for 10 consecutive time-steps and if all of them remain less than the threshold value of *G*_th_ = 1 × 10^−10^, we choose our *t*_*M*_. We find *t*_*M*_ for all seven sites and then choose the maximum value among them, thus we keep a single value of asymptotic limit (*t*_*M*_) for all seven-sites. By analyzing the gradients, we find the region of the trajectory, where the change in population of the site is very small. By knowing that, we keep the time-length of our trajectory *t*_*M*_ up to that region, because beyond *t*_*M*_ the change in population is very small, and ML is able to predict it. As the asymptotic limit for each trajectory is different, we have different values of *t*_*M*_ for each trajectory. In our training, we have included *t* → *∞*, corresponding to the asymptotic behavior at long-time. Using the strategy of different *t*_*M*_ for each trajectory allows us to include more sampling in our training set from hard-to-learn trajectories, while avoiding redundant sampling from easy-to-learn trajectories. For training, sampling is done with different training time-steps Δ*t*_train_ in different regions of the trajectory. We sample our training points from 0 ps–1 ps, 1 ps–1.5 ps, 1.5 ps–2.5 ps, 2.5 ps–5 ps, 5 ps–25 ps, 25 ps–50 ps, 50 ps–250 ps, 250 ps–*t*_*M*_ regions with Δ*t*_train_ = 5, 10, 25, 50, 100, 200, 500, 1000 fs, respectively. The number of training points depends on the number of trajectories N_traj_ chosen for training, training time-step Δ*t*_train_ and time-length of trajectories *t*_*M*_, which in turn depends on *G*_th_.

### Training architecture

We use convolutional neural network (CNN) architecture, because the importance of convolutional layers is much explored for image analysis, where these layers extract important features such as edges, textures, objects, and scenes. When it comes to time-series data, we are using convolutional layers in the hope to extract some important features from the data (such as the time influence). After learning those features, when we provide a test trajectory, the trained ML model will look for those features in that test trajectory^66^. Though we have used the CNN model, other neural network architectures such as long short-term memory (LSTM) is also an option. LSTM is considered to be more suitable for extracting long-time temporal dependencies in contrast to convolutional neural networks (CNNs) which are more local. However, CNNs are easy to train and in many studies, they have outperformed LSTM for future forecasting^67,68^.

We use 1000 trajectories as our training set TS and 200 trajectories as the validation set VS. After preparation of the input following Fig. 1, we build a CNN architecture and optimize it with hyperopt library^69^. The optimization was carried out only on 300 training trajectories from the training set TS. After optimization, our training architecture consists of two one-dimensional (1D) hidden convolutional layers, one maximum pooling layer, one flatten layer, three fully connected hidden dense layers and one output dense layer. The convolutional layers extract time-dependent correlations from a moving window, while maximum pooling layer pulls out the important information and decreases the size of the feature map which leads to reducing the computational cost. The flatten layer converts the output from the maximum pooling layer into 1D format as the fully connected dense layers, which are the traditional neural networks, can only work with 1D data. We train our CNN architecture using Keras software package^70^ with the TensorFlow in the backend^71^. Activation function, number of filters, kernel size and number of neurons for the respective convolutional and dense layers are given in Table 2. In our study, we train a single CNN model and with ca. 3.2 million training points and 900 epochs, training takes ca. 42 h on 32 Intel(R) Xeon(R) Gold 6226R CPUs @ 2.90 GHz. The optimized learning rate is 1 × 10^−3^ with adoptive mean optimizer and the batch size is 512. Using mean squared error function as a loss, we report 1.86 × 10^−7^ as the validation loss. The mean absolute error (MAE) and root mean square error (RMSE) averaged over 600 randomly chosen trajectories from the set of test trajectories STP (which were not part of the training process) are given in Table 1.

**Table 2:** Summary of the optimized neural network architecture with layers, output shape (OS), number of parameters (NP), activation function (AF), number of filters (NF), kernel size (KS), and number of neurons (NN).

Layers (type)	OS	NP	AF	NF	KS	NN
First hidden convolutional layer (1D)	(None, 103, 90)	360	relu	90	3	×
Second hidden convolutional layer (1D)	(None, 103, 70)	18,970	relu	70	3	×
Maximum pooling layer	(None, 51, 70)	0	×	×	×	×
Flatten layer	(None, 3570)	0	×	×	×	×
First hidden dense layer	(None, 512)	1,828,352	relu	×	×	512
Second hidden dense layer	(None, 512)	262,656	relu	×	×	512
Third hidden dense layer	(None, 512)	262,656	relu	×	×	512
Dense output layer	(None, 13)	6669	Linear	×	×	13

Total parameters: 2,379,663; trainable parameters: 2,379,663; non-trainable parameters: 0.

#### Input normalization and redundant time-functions

As we have multiple input elements, we need to normalize them all. In normalized input, we have $\lambda =\{{\lambda }_{1},{\lambda }_{2},{\lambda }_{3},\ldots, {\lambda }_{j}\}/{\lambda }_{\max }$, $\gamma =\{{\gamma }_{1},{\gamma }_{2},{\gamma }_{3},\ldots, {\gamma }_{k}\}/{\gamma }_{\max }$, and $T=\{{T}_{1},{T}_{2},{T}_{3},\ldots, {T}_{l}\}/{T}_{\max }$, where ${\lambda }_{\max }$, ${\gamma }_{\max }$, and ${T}_{\max }$ represent the maximum values of *λ*, *γ*, and *T*, respectively. We divide *n* = {*n*_1_, *n*_2_, *n*_3_, …, *n*_7_} = {1, 2, 3, …, 7} (labels corresponding to the seven rows in the reduced density matrix) by 10 to normalize their values, i.e., the input elements corresponding to the rows in the reduced density matrix are {0.1, 0.2, 0.3, …, 0.7}. Labels for sites with possible initial excitation are *m* = {0, 1}, which, respectively, represent initial excitation on site-1 and site-6. The input time is represented by a set of redundant time-functions $\left\{{f}_{i}(t)\right\}$, each of which is logistic function *f*(*t*) normalizing time. We use a set of 100 logistic functions ${f}_{k}(t)=1/(1+15\cdot \exp (-(t+{c}_{k})))$, where *c*_*k*_ = 5*k*−1.0 and *k* ∈ {0, 1, 2, …, 99}, i.e., each logistic function has the same shape and designed to cover the corresponding ≈ 5 ps region and is shifted with respect to the next logistic function by 5 ps, as shown in Supplementary Fig. 3. The infinity limit is given by all redundant time-functions set to one.

## Supplementary information

Supplementary Information

## Data Availability

Data can be re-generated using the script provided at https://github.com/Arif-PhyChem/AIQD_FMO.

## Code availability

The code is available at https://github.com/Arif-PhyChem/AIQD_FMO.
