# Supplementary material for: Predicting the future of excitation energy transfer in light-harvesting complex with artificial intelligence-based quantum dynamics
Source: Nat Commun. 2022 Apr 11;13:1930. doi: 10.1038/s41467-022-29621-w (PMC9001686; doi:10.1038/s41467-022-29621-w)
Supplement: Supplementary file 1 — Supplementary Information [file 41467_2022_29621_MOESM1_ESM.pdf]

Supplementary Information
Predicting the future of excitation energy transfer in light-harvesting complex with artificial intelligence-based quantum dynamics

Arif Ullah and Pavlo O. Dral

Supplementary Methods

The theory of local thermalising Lindblad equation of motion is well-documented in Ref. 1, however for the sake of completeness, we briefly outline it here. We begin from system Hamiltonian given in Eq. (2) of the main text, and rewrite it as

$$\mathbf{H}_{\mathrm{s}} = \sum_{i=1}^{n} \epsilon_i a_i^{\dagger} a_i + \sum_{j<i}^{n} J_{ij} \left(a_i^{\dagger} a_j + a_j^{\dagger} a_i \right) . \tag{1}$$

The $a_i^{\dagger}$ and a_i are the exciton creation and annihilation operators at site-i. Because of the fast EET, only single-excitation is considered, hence we have two possible states $|0\rangle$ and $|i\rangle = a_i^{\dagger}|0\rangle$. The exciton basis $|e\rangle = \sum_i c_i(e)|i\rangle$ is considered as eigenbasis of the Hamiltonian $\mathbf{H}_{\mathrm{s}}$, i.e., $\mathbf{H}_{\mathrm{s}}|e\rangle = \epsilon_i|e\rangle$. With the Born-Markov and secular approximations, the Lindblad master equation (to

second order in the system-bath coupling) for the dynamics of the reduced density matrix is written as

$$\frac{\partial \rho_{\rm s}(t)}{\partial t} = -\frac{i}{\hbar}\left[\mathbf{H}_{\rm s} + \mathbf{H}_{\rm reorg}, \rho_{\rm s}(t)\right] + L_{\rm env}(\rho_{\rm s}(t)) + L_{\rm s-env}(\rho_{\rm s}(t)), \tag{2}$$

where the Lindblad superoperators $L_{\rm env}$ and $L_{\rm s-env}$ are given by (k = env, s − env)

$$L_k(\rho_{\rm s}) = \sum_{\omega}\sum_{i,j}\kappa^k_{ij}(\omega)\left[A^k_i(\omega)\rho_{\rm s}A^{k\dagger}_j(\omega) - \frac{1}{2}A^k_i(\omega)A^{k\dagger}_j(\omega)\rho - \frac{1}{2}\rho_{\rm s}A^k_i(\omega)A^{k\dagger}_j(\omega)\right].$$

In local thermalising approach, the corresponding Lindblad generators for $\mathbf{H}_{\rm env}$ are

$$A^{\rm env}_i(\omega) = \sum_{\omega-\omega'} c^*_i\left(e_\omega\right)c_i\left(e_{\omega'}\right)\left|e_\omega\right\rangle\left\langle e_{\omega'}\right|, \tag{3}$$

where the summation is over all transitions between eigenstates $|e_\omega\rangle$ and $|e_{\omega'}\rangle$ with frequency ω. The $c^*_i\left(e_\omega\right)c_i\left(e_{\omega'}\right)$ is a factor weighting transfer between the two eigen states $|e_\omega\rangle$ and $|e_{\omega'}\rangle$. The $c_i\left(e_\omega\right)$ and $c_i\left(e_{\omega'}\right)$ are the ith site coefficient in eigenstate $|e_\omega\rangle$ and $|e_{\omega'}\rangle$, respectively. The rate κ in Eq.(2) is considered site-independent with the following expression

$$\gamma^{\rm env} = 2\pi\left[J(\omega)(1+n(\omega)) + J(-\omega)n(-\omega)\right] \tag{4}$$

where $J(\omega)$ is spectral density and $n(\omega) = 1/\left[\exp\left(\frac{\hbar\omega}{k_{\rm B}T}\right) - 1\right]$ is bosonic distribution.

For $\mathbf{H}_{\rm s-env}$, $A^{\rm s-env}_i(\omega_e) = c_i(e)|0\rangle\langle e|$ where $\hbar\omega_e$ is the molecular transition frequency. The respective rate $\kappa^{\rm s-env}_{ij}(\omega)$ is diagonal $\kappa^{\rm s-env}_{ij}(\omega) = \delta_{ij}\kappa^{\rm s-env}_{ij}(\omega)$

and site-independent $\kappa_{ii}^{\mathrm{s-env}}(\omega) = \kappa^{\mathrm{s-env}}(\omega)$. The $\mathbf{H}_{\mathrm{reorg}}$ is divided into two parts $\mathbf{H}_{\mathrm{reorg}}^{\mathrm{env}} + \mathbf{H}_{\mathrm{reorg}}^{\mathrm{s-env}}$ where $\mathbf{H}_{\mathrm{reorg}}^{k} = \Sigma_{\omega,i,j} S_{ij}^{k}(\omega) A_i^{k\dagger}(\omega) A_j^{k}(\omega)$ (for $k = \mathrm{env}, \mathrm{s-env}$) with $S_{ij}(\omega)$ as the imaginary part of the half-sided Fourier transform of the bath correlation function.

Supplementary Figures

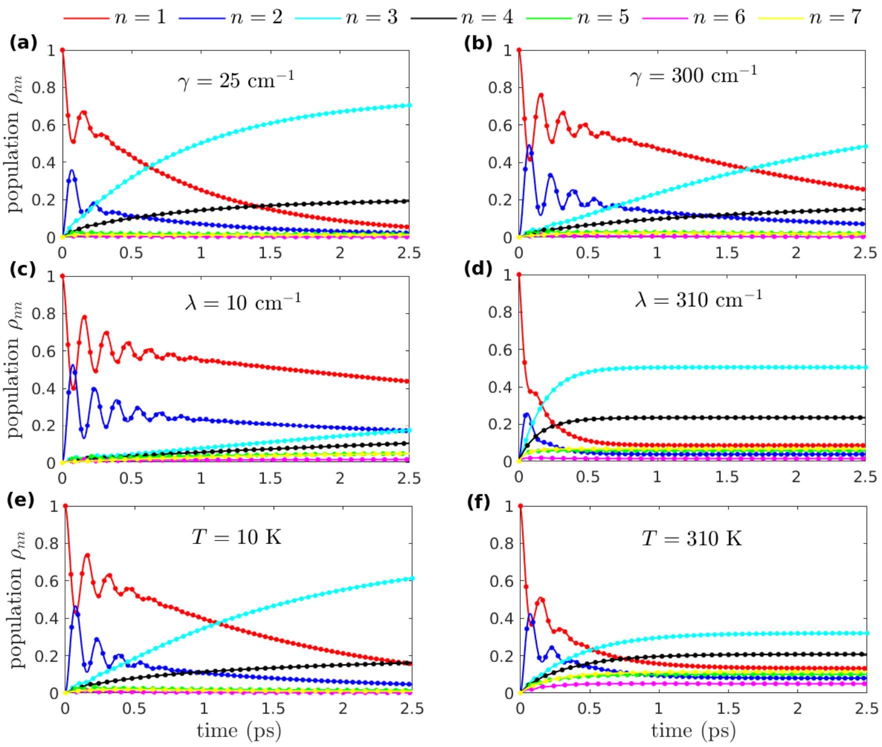

Supplementary Figure 1: Population of the seven sites in the FMO complex as a function of time. The results are shown for extreme values of γ (as a scale of non-Markovian character of the bath), λ (as a scale of decoherence strength) and T in our parameter space with the aim to see their effect on the accuracy (see Supplementary Table 1). Parameters are (a) $\gamma = 25$, $\lambda = 100$, $T = 50$, (b) $\gamma = 300$, $\lambda = 100$, $T = 50$, (c) $\gamma = 100$, $\lambda = 10$, $T = 150$, (d) $\gamma = 100$, $\lambda = 310$, $T = 150$, (e) $\gamma = 175$, $\lambda = 100$, $T = 30$ and (f) $\gamma = 175$, $\lambda = 100$, $T = 310$. The initial excitation is considered on site-1. The results of AI-QD are compared to the results of LTLME-QD (dots). γ and λ are in the units of cm^{-1}, while T is in the units of K.

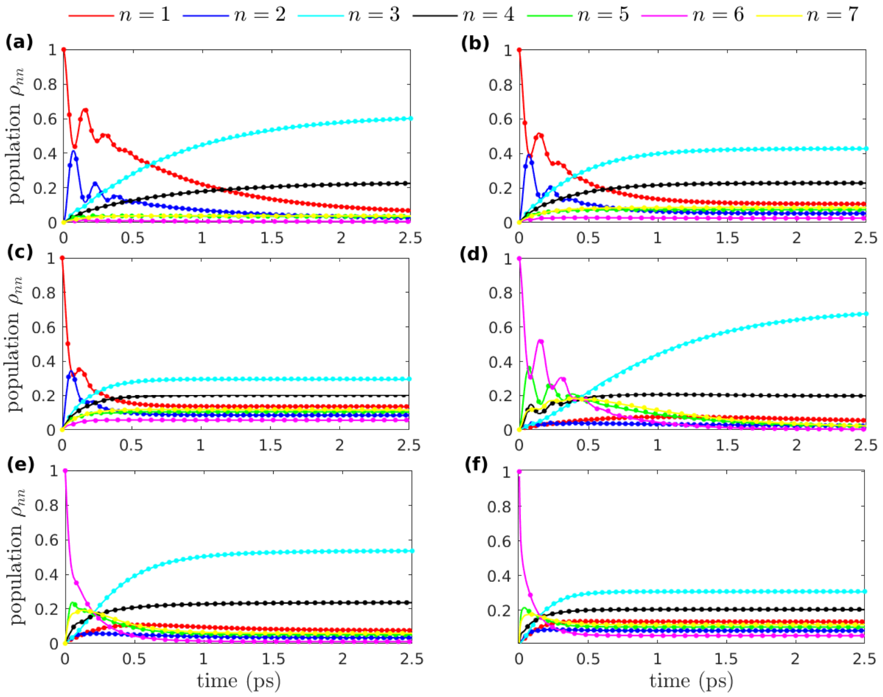

Supplementary Figure 2: Population of the seven sites in the FMO complex as a function of time. The results are shown for parameters which do not appear in the training set at all. In (a), (b) and (c), the initial excitation is considered on site-1. Other parameters are (a) $\gamma = 80$, $\lambda = 85$, $T = 100$, (b) $\gamma = 205$, $\lambda = 185$, $T = 195$ and (c) $\gamma = 350$, $\lambda = 350$, $T = 350$. In (d), (e) and (f), the initial excitation is on site-6 and other parameters are (d) $\gamma = 65$, $\lambda = 35$, $T = 55$, (e) $\gamma = 155$, $\lambda = 145$, $T = 135$ and (f) $\gamma = 330$, $\lambda = 330$, $T = 330$. The results of AI-QD are compared to the results of LTLME-QD (dots). γ and λ are in the units of cm^{-1}, while T is i the units of K. The respective errors are given in Supplementary Table 2. Note that (c) and (f) are outside the range of parameters used for training.

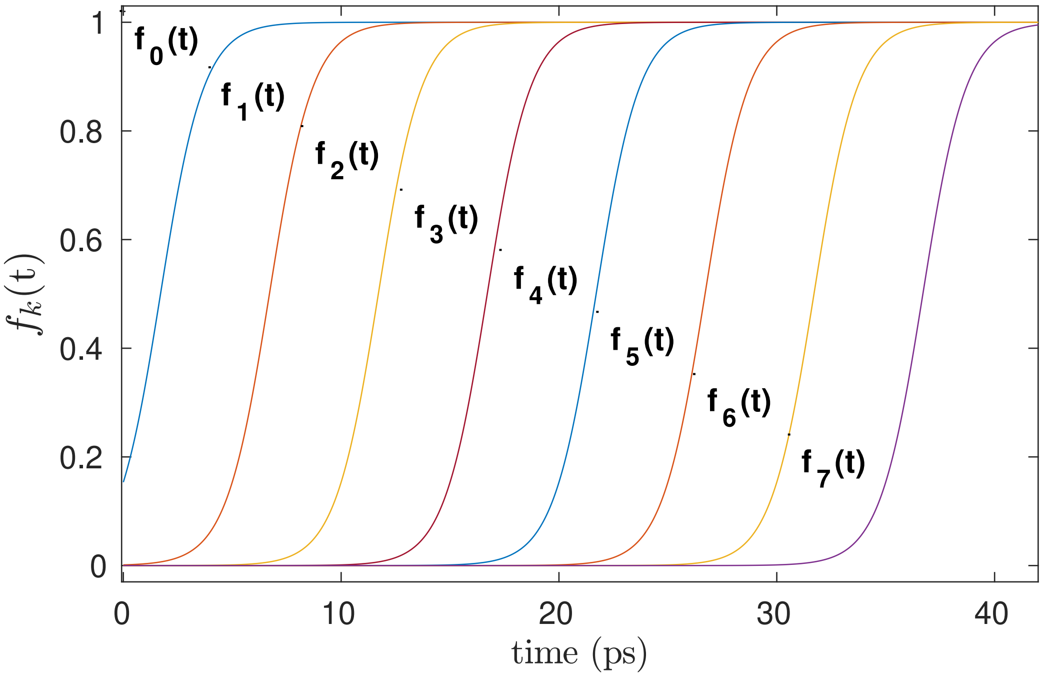

Supplementary Figure 3: Logistic functions $f_k(t) = 1/(1+15 \cdot \exp(-(t+c_k)))$, where $c_k = 5k - 1.0$ and $k \in \{0, 1, 2, \ldots, 99\}$. Each logistic function is designed to cover the corresponding ≈ 5 ps region. Here only the first eight logistic functions are shown covering a time-region of 40 ps.

Supplementary Tables

Supplementary Table 1: Mean absolute error (MAE) and root mean square error (RMSE) for the test trajectories presented in Supplementary Fig. 1. Here we show the dependence of accuracy on parameters γ, λ and T. The test trajectories were propagated up to 1 ns. $\mathcal{R}\{\rho_{mn,\, n\neq m}\}$ and $\mathcal{I}\{\rho_{mn,\, n\neq m}\}$ respectively represent the real and imaginary part of the off-diagonal terms.

parameters				mean absolute error			root mean square error		
IES	γ	λ	T	ρ_{nn}	$\mathcal{R}\{\rho_{mn,\, n\neq m}\}$	$\mathcal{I}\{\rho_{mn,\, n\neq m}\}$	ρ_{nn}	$\mathcal{R}\{\rho_{mn,\, n\neq m}\}$	$\mathcal{I}\{\rho_{mn,\, n\neq m}\}$
1	25	100	50	$8.7\cdot10^{-4}$	$9.0\cdot10^{-4}$	$3.6\cdot10^{-4}$	$1.2\cdot10^{-3}$	$1.5\cdot10^{-3}$	$6.4\cdot10^{-4}$
1	300	100	50	$4.6\cdot10^{-4}$	$2.5\cdot10^{-4}$	$1.3\cdot10^{-4}$	$6.8\cdot10^{-4}$	$3.7\cdot10^{-4}$	$1.9\cdot10^{-4}$
6	25	100	50	$8.3\cdot10^{-4}$	$7.1\cdot10^{-4}$	$2.3\cdot10^{-4}$	$1.1\cdot10^{-3}$	$1.1\cdot10^{-3}$	$3.5\cdot10^{-4}$
6	300	100	50	$6.7\cdot10^{-4}$	$3.0\cdot10^{-4}$	$1.4\cdot10^{-4}$	$1.0\cdot10^{-4}$	$4.4\cdot10^{-4}$	$2.0\cdot10^{-4}$
1	100	10	150	$6.5\cdot10^{-4}$	$2.8\cdot10^{-4}$	$1.3\cdot10^{-4}$	$1.3\cdot10^{-3}$	$5.0\cdot10^{-4}$	$1.9\cdot10^{-4}$
1	100	310	150	$2.3\cdot10^{-3}$	$1.0\cdot10^{-3}$	$3.1\cdot10^{-4}$	$3.6\cdot10^{-3}$	$1.7\cdot10^{-3}$	$5.1\cdot10^{-4}$
6	100	10	150	$7.4\cdot10^{-4}$	$2.8\cdot10^{-4}$	$1.6\cdot10^{-4}$	$1.4\cdot10^{-3}$	$5.1\cdot10^{-4}$	$2.5\cdot10^{-4}$
6	100	310	150	$1.6\cdot10^{-3}$	$6.8\cdot10^{-4}$	$2.3\cdot10^{-4}$	$2.6\cdot10^{-3}$	$1.0\cdot10^{-3}$	$3.5\cdot10^{-4}$
1	175	100	30	$4.3\cdot10^{-4}$	$3.5\cdot10^{-4}$	$1.4\cdot10^{-4}$	$6.3\cdot10^{-4}$	$5.8\cdot10^{-4}$	$2.0\cdot10^{-4}$
1	175	100	310	$1.6\cdot10^{-3}$	$4.7\cdot10^{-4}$	$2.0\cdot10^{-4}$	$2.5\cdot10^{-3}$	$7.5\cdot10^{-4}$	$2.9\cdot10^{-4}$
6	175	100	30	$5.1\cdot10^{-4}$	$3.0\cdot10^{-4}$	$1.4\cdot10^{-4}$	$8.5\cdot10^{-4}$	$4.9\cdot10^{-4}$	$2.2\cdot10^{-4}$
6	175	100	310	$1.3\cdot10^{-3}$	$3.8\cdot10^{-4}$	$2.0\cdot10^{-4}$	$2.1\cdot10^{-3}$	$6.3\cdot10^{-4}$	$3.0\cdot10^{-4}$

Supplementary Table 2: Mean absolute error (MAE) and root mean square error (RMSE) for the test trajectories presented in Supplementary Fig. 2. The test trajectories were propagated up to 1 ns. $\mathcal{R}\{\rho_{mn,\, n\neq m}\}$ and $\mathcal{I}\{\rho_{mn,\, n\neq m}\}$ respectively represent the real and imaginary part of the off-diagonal terms.

parameters				mean absolute error			root mean square error		
IES	γ	λ	T	ρ_{nn}	$\mathcal{R}\{\rho_{mn,\, n\neq m}\}$	$\mathcal{I}\{\rho_{mn,\, n\neq m}\}$	ρ_{nn}	$\mathcal{R}\{\rho_{mn,\, n\neq m}\}$	$\mathcal{I}\{\rho_{mn,\, n\neq m}\}$
1	80	85	100	$9.5\cdot10^{-4}$	$4.5\cdot10^{-4}$	$1.9\cdot10^{-4}$	$1.5\cdot10^{-3}$	$7.0\cdot10^{-4}$	$2.6\cdot10^{-4}$
1	205	185	195	$1.2\cdot10^{-3}$	$4.5\cdot10^{-4}$	$2.0\cdot10^{-4}$	$2.1\cdot10^{-3}$	$7.4\cdot10^{-4}$	$2.9\cdot10^{-4}$
1	350	350	350	$4.9\cdot10^{-3}$	$1.2\cdot10^{-3}$	$5.0\cdot10^{-4}$	$6.9\cdot10^{-3}$	$1.6\cdot10^{-3}$	$5.8\cdot10^{-4}$
6	65	35	55	$7.8\cdot10^{-4}$	$4.4\cdot10^{-4}$	$2.1\cdot10^{-4}$	$1.2\cdot10^{-3}$	$6.2\cdot10^{-4}$	$3.4\cdot10^{-4}$
6	155	145	135	$1.1\cdot10^{-3}$	$5.0\cdot10^{-4}$	$1.9\cdot10^{-4}$	$1.9\cdot10^{-3}$	$7.5\cdot10^{-4}$	$2.8\cdot10^{-4}$
6	330	330	330	$3.8\cdot10^{-3}$	$9.0\cdot10^{-4}$	$5.3\cdot10^{-4}$	$5.5\cdot10^{-3}$	$1.2\cdot10^{-3}$	$7.6\cdot10^{-4}$

Supplementary References

[1] Mohseni, M., Rebentrost, P., Lloyd, S. & Aspuru-Guzik, A. Environment-assisted quantum walks in photosynthetic energy transfer. *The Journal of Chemical Physics* **129** (17), 174106 (2008).
